# The Effects of Chinese Medicine QRD, Antibiotics, and Probiotics on Therapy and Gut Microbiota in Septic Rats

**DOI:** 10.3389/fcimb.2021.712028

**Published:** 2021-10-14

**Authors:** Huiling Cao, Chunhui Zong, Wenkui Dai, Qiaoying Gao, Donghua Li, Xianzhong Wu, Dongfang Li, Yi-Wei Tang, Shangwei Wu

**Affiliations:** ^1^ Department of Gynecology and Obstetrics, Tianjin Medical University General Hospital, Tianjin, China; ^2^ Institute of Acute Abdominal Diseases, Tianjin Hospital of Integrated Chinese and Western Medicine, Tianjin, China; ^3^ Department of Obstetrics and Gynecology, Peking University Shenzhen Hospital, Shenzhen, China; ^4^ Institute of Obstetrics and Gynecology, Shenzhen Peking University- the Hong Kong University of Science and Technology Medical Center, Shenzhen, China; ^5^ Shenzhen Key Laboratory on Technology for Early Diagnosis of Major Gynecologic Diseases, Peking University Shenzhen Hospital, Shenzhen, China; ^6^ Department of Microbial Research, WeHealthGene Institute, Shenzhen, China; ^7^ Department of Medical Affairs, Danaher Diagnostic Platform/Cepheid (China), Shanghai, China; ^8^ Department of Clinical Microbiology, Tianjin KingMed Center for Clinical Laboratory Co. Ltd., Tianjin, China

**Keywords:** sepsis, gut microbiota, antibiotics, probiotics, Qing Re Jie Du Fang Decoction

## Abstract

Sepsis is a common and often treacherous medical emergency with a high mortality and long-term complications in survivors. Though antibiotic therapy can reduce death rate of sepsis significantly, it impairs gut microbiota (GM), which play imperative roles in human health. In this study, we compared the therapeutic effects of antibiotics, probiotics, and Chinese medicine QRD on the survival rates of septic model and observed the GM characteristics of experimental rats *via* 16S rRNA gene amplicon sequencing. The 72 h survival rates of septic rat demonstrated the significant therapeutic effects in the three groups treated with antibiotics (AT), Chinses medicine QRD (QT), and probiotics (PT), which were elevated from the survival rate of 26.67% for the sepsis control group (ST) to 100.0% for AT, 88.24% for QT, and 58.33% for PT. The original characteristics of GM identified in the sham operation controls (SC) were relatively similar to those in PT and QT; nevertheless, the AT rats were shown dramatically decreased in the GM diversity. In addition, the septic rats in AT were revealed the higher abundances of *Escherichia Shigella*, *Proteus, Morganella*, *Enterococcus*, and *Lysinibacillus*, but the lower those of *Parabacteroides, Alistipes*, *Desulfovibrio*, *Bacteroides*, *Helicobacter*, *Mucispirillum*, *Oscillibacter*, *Lachnospiraceae*, and *Ruminiclostridium 9*, when compared to the PT and QT rats. By contrast, the GM of PT and QT rats shared similar diversity and structure. Our findings indicated that QRD increased the survival rates without impairment of the GM characteristics, which provides novel insights into the role of Chinese medicine in therapy and long-term recovery of sepsis.

## Introduction

Sepsis is a life-threatening syndrome caused by dysregulated host responses to infection and can result in tissue damage, organ failure, and even death (Kim and Choi, 2020). In the United States, the cost of sepsis management ranks the top of the hospitalization charges among all diseases (Paoli et al., 2018). Though onset cases are increasingly reported, the mortality of sepsis decreased significantly due to in-time therapy. Nevertheless, it is clearly recognized that the survivals of sepsis suffer from a long period of physical, psychological, and cognitive disabilities, along with progressively declining life quality and increasing death risk (Cecconi et al., 2018; Barichello et al., 2019).

GM has been considerably noticed due to its roles on intact gut barrier and host physiological health, which is associated with sepsis pathogenesis and prognosis. An increasing number of studies revealed sepsis-associated GM dysbiosis, which might contribute to the development of organ failure (Haak and Wiersinga, 2017; Coopersmith et al., 2018; Haak et al., 2018). Taft et al. also observed GM imbalance in preterm infants with late-onset (LOS) sepsis (Taft et al., 2015). In addition, Prescott group demonstrated that GM imbalance increased the risk of sepsis (Prescott et al., 2015). Antibiotic therapy, which is one of the most important agents in the septic management, can impair GM significantly and then influence the long-term prognosis (Ojima et al., 2016; Haak et al., 2018).

The Chinese medicine Qing Re Jie Du Fang Decoction (QRD), composed of Dahuang (*Rheum palmatum* L.), Huangqin (*Scutellaria baicalensis* Georgi), Baitouweng (*Pulsatilla chinensis* Regel), and Baijiangcao (*Patrinia scabiosaefolia*), has been broadly used for heat-clearing and detoxification according to Medical Treasures of the Golden Chamber. In addition, QRD is commonly prescribed to treat the patients with suppurative appendicitis, intra-abdominal infections, and even multiple organ dysfunction syndrome (MODS). In this study, we aimed to elucidate the clinical effect and GM alteration among the septic rats in AT, PT, and QT. It is expected that this effort will provide extensive insights into alternative therapies of sepsis.

## Materials and Methods

### Experimental Procedure

The animal experiments were performed in accordance with the principle of “Regulations of Experimental Animal Administration” issued by the State Committee of Science and Technology of the People’s Republic of China. The procedures were approved by the Animal Care and Use Committee of Tianjin Hospital of Integrated Chinese and Western Medicine, and most of the experimental details were described as previously published (Liu et al., 2013a). Male Wistar rats aged between 8 and 9 weeks were purchased from the Animal Center of Chinese Military Medical Academy (License number: SCXK-[Army] 2014-0001). Acclimatization was given to the rats for a week as previously described (Liu et al., 2013a). In this study, all the male rats with 8–9 weeks age were randomly allotted into five groups: sham operation control (SC, n=12); septic rats without treatment (ST, n=30); septic rats receiving antibiotics (AT, n=12); septic rats with probiotics (PT, n=24), and septic rats receiving QRD (QT, n=17). Then the sham operation or CLP (cecal ligation and puncture) was performed according to the experimental requirement for each group. Rats in SC and ST were given sterile water in 12 ml/kg, while the AT, PT, and QT groups were treated by the cefaclor sustained-release capsules (100 mg/kg, Yangtze River Pharmaceutical Co., Ltd.) and metronidazole (120 mg/kg, Tianjin Pacific Pharmaceutical Co., Ltd.), probiotics (400 mg/kg, Golden Bifid, Inner Mongolia Shuangsi Pharmaceutical Co., Ltd.), and QRD in the dose of 24 g/kg crude drugs, respectively. The QRD prescription consisted of *Rheum palmatum* L. for laxative, *Scutellaria baicalensis* Georgi for clearing dampness and purging fire, *Pulsatilla chinensis* Regel for stopping dysentery, and *Patrinia scabiosaefolia* for discharging pus and eliminating congestion; the four herbs all have the therapeutic properties of clearing heat and detoxification. The QRD reagent consisted of the four components in the ratio of 2:2:3:3 and was made into 2 g/ml crude drug according to previous studies (Liu et al., 2013b). Rats in AT, PT, and QT group received treatment once a day for 3 days, while sterile water was given to the rats in SC and ST group. Survival rate was the ratio of the number of survived rats after 72 h to total number of rats in each group.

### Sample Collection

The contents of cecum base were collected from the dying rats of ST group and the survivors of the other groups; the latter were killed by cervical dislocation after 72 h of CLP surgery, then the 72 h survival rates of the five groups were recorded. Microbial samples were collected by squeezing the feces out of the cecum, and placed in prelabeled sterile conical tubes and then immediately stored at −80°C for further processing.

### Genomic DNA Extraction and 16S rRNA Gene Amplicon Sequencing

Ten samples from each group were randomly chosen to conduct sequencing, which were respectively labeled Z1~Z10 for group SC, S1~S10 for ST, P1~P10 for AT, Y1~Y10 for PT, and D1~D10 for QT. Microbial DNAs were extracted from the cecum contents by TIANamp Stool DNA Kit (TIANGEN BIOTECH CO., LTD, China) according to the manufacturer’s protocol. The V3 hypervariable regions of 16S rRNA gene were then amplified using the pair of forward (341F 5’-CCTACGGGAGGCAGCAG-3’) and reverse primer (518R 5’-ATTACCGCGGCTGCTGG -3’), and sequenced by Ion Torrent PGM platform (Life Technologies, Carlsbad, CA, USA). Raw data have been uploaded into NCBI database (accession number is PRJNA764854).

### Statistical Analysis and Visualization

Filtered reads, produced by FASTX Toolkit (version 0.0.13), were further processed by QIIME: connected into tags and then clustered into operational taxonomic units (OTUs) with 97% similarity. The representative OTUs were aligned to the SLIVA reference database (Release123) for analyzing bacterial components in each sample. The calculation of Shannon index and non-metric multidimensional scaling (NMDS), which represent α-diversity and β-diversity of GM, respectively, were analyzed using the “Vegan” package in R (version 3.6.3). Wilcoxon rank-sum test was utilized to analyze intergroup differences, which were adjusted by the false discovery rate (FDR). All graphs were depicted through R software (version 3.6.3).

## Results

### Survival Rate in Each Group

All rats in the SC group survived, and survival rate of septic rats in the ST group was 26.67% (**Table 1**). The three therapies significantly increased the survival rates of septic rats when compared to the ST group (*P*<0.05): 100% for AT group, 58.33% for PT, and 88.24% for QT (**Table 1**). In addition, the survival rate of PT rats was significantly lower than those of AT and QT (*P*<0.05), while an insignificant difference occurred between AT and QT group (*P*=0.335) (**Table 1**).

**Table 1:** Survival rate in each group.

Group	Survival	Death	Total	Survival rats
SC	12	0	12	100.00%
ST	8	22	30	26.67%
AT	12	0	12	100.00%
PT	14	10	24	58.33%
QT	15	2	17	88.24%

The survival rates were compared between each treated group (AT, PT, and QT) and untreated control group (ST), and every two treated groups using Fisher’s exact probability (one-tailed probability).

ST compared to AT, P =0.000; ST compared to PT, P =0.019; ST compared to QT, P =0.000; AT compared to PT, P =0.008; AT compared to QT, P =0.335; PT compared to QT, P =0.039.

Obvious differences were accepted at p values of <0.05.

### Sham Operation Control and Septic Rats Harbor Different GM

**Figure 1:**
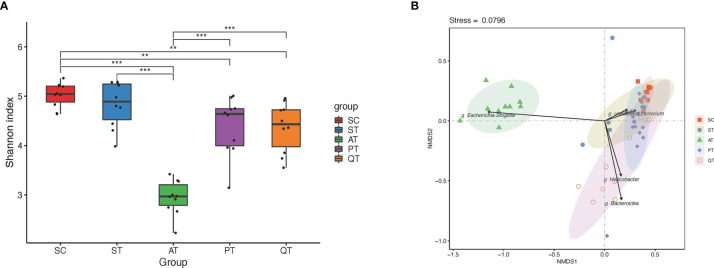
Microbiota diversity and Non-metric multidimensional scaling (NMDS) distribution in five groups. **(A)** The microbial diversity in the AT group was significantly lower than that in the PT and QT groups. **(B)** NMDS analysis, the gut microbiota samples from AT group showed an individual feature and separated far from that of the other groups, while the other groups shared partly with each other. **, and *** stand for the FDR-value <0.01, and <0.001, respectively.

**Figure 2:**
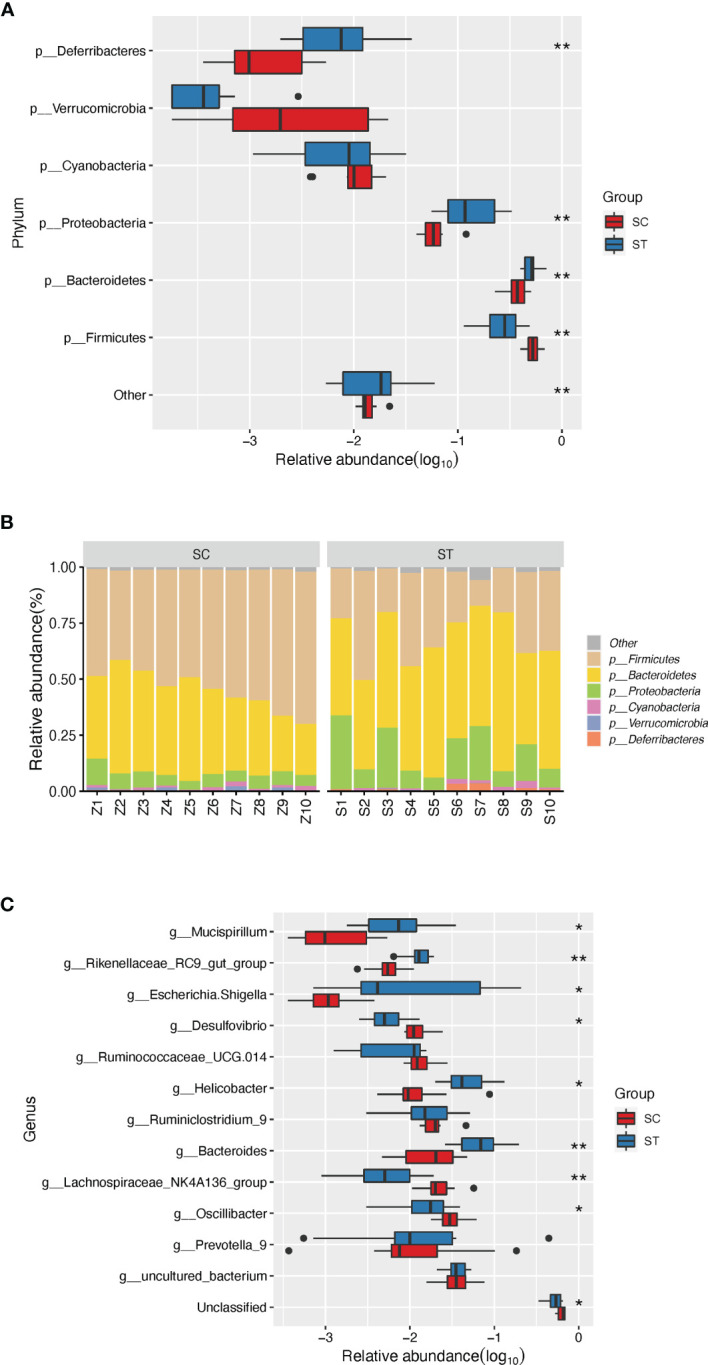
The discrepant phyla (relative abundance of top 5) and genera (relative abundance of top 10) between SC and ST group. **(A)** Four distinct phyla were found between SC and ST group. **(B)** The barplot of composition of microbial community in SC and ST group. **(C)** Eight discrepant genera were sought out between SC and ST groups. *, and ** stand for the FDR-value <0.05, and <0.011, respectively.

Data evaluation indicated qualified sequencing reads for bacterial classification (**Table 2** and **Supplementary Table S1**). Analysis results showed no significant difference of GM diversity between SC and ST (**Figure 1A**). Non-metric multidimensional scaling (NMDS) analysis also indicated that the microbial samples in SC and ST were clustered together (**Figure 1B**). As GM components, the relative abundances of four phyla were statistically different between the two groups (**Figures 2A, B**): *Deferribacteres* (SC *versus* ST, 0.21 ± 0.21% *versus* 1.18 ± 1.24%, *P <*0.01, FDR <0.01)*, Proteobacteria* (6.23 ± 2.21% *versus* 15.59 ± 9.68%, *P <*0.01, FDR <0.01), *Bacteroidetes* (36.98 ± 8.98% *versus* 50.86 ± 9.17%, *P <*0.01, FDR <0.01), and *Firmicutes* (53.37 ± 8.85% *versus* 29.26 ± 11.78%, *P <*0.001, FDR <0.01).

**Table 2:** Assessment of sequencing data.

Group	Tag (Range)	OTU (Range)	Genus no.
SC	69,857 ± 61,833 (6,860–137,941)	719 ± 78 (576–819)	53 ± 1
ST	109,308 ± 29,420 (54,436–139,858)	723 ± 57 (622–803)	48 ± 11
AT	99,363 ± 22,801 (61,698–123,517)	561 ± 58 (485–662)	36 ± 10
PT	45,279 ± 56,213 (6,360–136,579)	529 ± 119 (297–678)	46 ± 6
QT	38,101 ± 51,098 (5,864–121,204)	548 ± 96 (318–665)	53 ± 8

At genus level, eight of the top 10 abundant genera were differentially enriched in the two groups. Compared to the SC, the ST rats had higher abundances of *Mucispirillum* (1.14 ± 1.19%, *P <*0.01, FDR <0.05), *Rikenellaceae RC9 gut group* (1.31 ± 0.45%, *P <*0.001, FDR <0.01), *Escherichia Shigella* (4.97 ± 7.61%, *P <*0.01, FDR <0.05), *Helicobacter* (5.47 ± 3.63%, *P <*0.01, FDR <0.05), and *Bacteroides* (7.96 ± 5.29%, *P <*0.001, FDR <0.01) in GM. In contrast, the higher proportions of *Desulfovibrio* (1.27 ± 0.50%, *P <*0.01, FDR <0.05), *Lachnospiraceae NK4A136 group* (2.46 ± 1.28%, *P <*0.001, FDR <0.01), and *Oscillibacter* (3.22 ± 1.24%, *P <*0.05, FDR <0.05) were detected in the SC group (**Figure 2C**).

### Antibiotic, Probiotic, and QRD Therapy Impose Distinct Effects on GM of Septic Rats

Based on NMDS analysis, the microbial samples in AT group were notably separated from those in other four groups (**Figure 1B**). Microbes in QT group were sparsely distributed, representing a group-specific feature of inter-individual differences, while the microbial distribution in PT rats was shown closer to that in SC group (**Figure 1B**). Additionally, the AT rats showed a dramatic decrease of GM diversity when compared to the PT and QT rats (**Figure 1A**).

**Figure 3:**
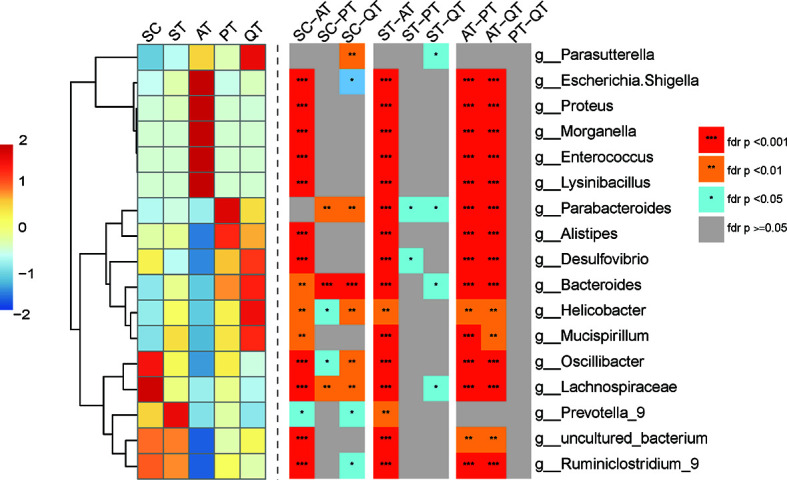
Heatmap of top seven species abundance and compositional differences of cecum content from five treated groups across the genus level. (Left) Abundance distribution of dominant 16 genera (Y-axis) across SC, ST, AT, PT, and QT groups (X-axis) were displayed, which were arranged along the x-axes based on hierarchical clustering. Red represents the genera with high abundance in the corresponding treated groups, while blue represents genera with low abundance. (Right) Heatmap depicting differentially abundant genera of any two group of five treated groups. FDR values indicated by asterisks on the color lump of Figure (***FDR < 0.001, **FDR < 0.01, *FDR < 0.05).

At genus level, nearly no difference was detected in the most abundant genera between PT and QT group (**Figure 3**). Nevertheless, the rats in AT group had significantly different GM structure as compared to PT and QT group, including the elevated abundances of *Escherichia Shigella* (AT *versus* PT *versus* QT, 41.06 ± 12.30% *versus* 0.70 ± 0.86% *versus* 3.84 ± 4.15%), *Proteus* (4.93 ± 4.01% *versus* 0.01 ± 0.03% *versus* 0.01 ± 0.02%)*, Morganella* (7.42 ± 5.49% *versus* 0.00 ± 0.01% *versus* 0.05 ± 0.08%), *Enterococcus* (9.07 ± 3.40% *versus* 0.01 ± 0.02% *versus* 0.01 ± 0.01%), and *Lysinibacillus* (7.51 ± 6.65% *versus* 0.00 ± 0.00% *versus* 0.03 ± 0.09%) (FDR <0.001), and the decreased proportions of *Parabacteroides* (0.03 ± 0.04% *versus* 2.18 ± 2.94% *versus* 1.12 ± 0.63%)*, Alistipes* (0.04 ± 0.04% *versus* 1.72 ± 1.69% *versus* 1.35 ± 2.39%), *Desulfovibrio* (0.04 ± 0.06% *versus* 1.49 ± 0.52% *versus* 1.89 ± 2.15%), *Bacteroides* (0.31 ± 0.63% *versus* 16.16 ± 14.38% *versus* 20.33 ± 10.62%)*, Helicobacter* (0.87 ± 2.51% *versus* 5.93 ± 5.39% *versus* 10.49 ± 10.88%)*, Mucispirillum* (0.01 ± 0.03% *versus* 1.12 ± 1.09% *versus* 1.72 ± 3.43%)*, Oscillibacter* (0.02 ± 0.02% *versus* 1.94 ± 0.83% *versus* 0.95 ± 0.88%), *Lachnospiraceae* (0.00 ± 0.01% *versus* 0.61 ± 0.74% *versus* 0.11 ± 0.10%), and *Ruminiclostridium 9* (0.10 ± 0.20% *versus* 1.46 ± 0.92% *versus* 1.09 ± 0.55%) (FDR <0.01). In addition, the microbial samples in AT group showed significant difference in GM characteristics from SC and ST group, while QRD and probiotics had less impact on GM (**Figure 3**).

## Discussion

Sepsis, one of the life-threatening diseases worldwide, might be characterized by dysbiosis of gut microenvironment, including the gut leak and intrusion of intestinal microbes to bloodstream. Wan et al. reported significantly lower bacterial diversity in septic shock patients compared with healthy subjects (Wan et al., 2018). In another study (Lankelma et al., 2017), the assessment of the fecal composition from 34 patients admitted to the intensive care unit exhibited the reduction of bacterial diversity in their GMs. Similarly, the data in this study showed that the septic rats of ST group possessed the decreased GM diversity than the non-sepsis controls, despite the difference was not statistically significant probably because of the relative small sample scale. Additionally, the phylum *Proteobacteria*, a potential signature of dysbiosis in intestinal microiota (Shin et al., 2015), was also shown the significant increase in the ST rats. The GM dysbiosis of sepsis may result from infections or a wide variety of intervention (Lankelma et al., 2017; Wan et al., 2018). For instance, some enriched genera identified in the GM of septic rats in this study, such as *Escherichia Shigella* and *Helicobacter*, were actually well-known infectious agents (Komatsu et al., 2016; Li et al., 2017).

Antimicrobial agents can kill and inhibit the intestinal and intra-abdominal pathogenic microbes in a short time, which may explain the high survival rate of the septic rats under an in-time antibiotic therapy. Nevertheless, antibiotics also impair some of the normal GM colonizers (Zhang and Chen, 2019), which play key roles in keeping an intact intestinal barrier that is pivotal in protecting against sepsis (Takiishi et al., 2017). Probiotics have the potential to promote intestinal health, but a time-consuming process would be necessary to colonize in the gut lumen firstly then inhibit pathogens and restore the impaired intestinal barrier (Toscano et al., 2017). Thus, a probiotic therapy was generally required a course of longer than 2 weeks for the treatment of chronic diseases (Lewis-Mikhael et al., 2020; Oniszczuk et al., 2021). This probiotic mechanism may partly explain the lower survival rate of PT rats due to the short period time of the 72 h experiment.

In recent years, Chinese medicine is drawing attention in modulating GM and mitigating symptoms in some diseases (Tong et al., 2018; Jia et al., 2020), through metabolism or immune regulation (Yu et al., 2019). Our earlier investigations demonstrated the therapeutic effect of QRD on several acute abdominal syndromes and sepsis (Liu et al., 2013b). This study explored that the survival rate of QT rats was only second to that of septic rats treated with antibiotics, the tremendous effect of QRD on the experimental animal was simultaneously along with relatively less GM alteration, which obviously differed from the corresponding phenomenon observed in AT group. It indicated that QRD could treat the sepsis efficiently but barely impair the GM function. Several studies provided the evidence that QRD could improve intestinal barrier integrity and protect host against sepsis (Cui et al., 2019; Wu et al., 2019; Ji et al., 2020; Cui et al., 2021; Hua et al., 2021). Together with other findings, this study provides an additional insight into sepsis therapy *via* Chinese medicine.

Although this study implies the potential effects of QRD in treatment of sepsis and maintenance of GM balance, the specific mechanism could not be elucidated by several limitations. Firstly, the sample scale in each group and the observation time for long-term effects of therapy agents. Secondly, analytical scope and depth of the16S rRNA sequencing platform for key bacterial players and associated mechanisms at strain level. Thirdly, shortage of the approaches to reveal fungi and virus, which are also key players in gut microenvironment. In future investigations, metagenomic and metabolomics would be preferentially applied to unravel microbe-microbe and microbe-host interactions during QRD treatment, and then unmask mechanisms of QRD therapy.

## Data Availability Statement

The data presented in the study are deposited in the NCBI, accession number: PRJNA764854.

## Ethics Statement

The animal study was reviewed and approved by the Animal Care and Use Committee of Tianjin Hospital of Integrated Chinese and Western Medicine.

## Author Contributions

SW and Y-WT formed the hypothesis and designed the project. XW established the fundamental approach for this field. SW and XW funded the project. HC, CZ, QG, and DHL performed the experiments, data collection, and preliminary sorting. WD and DFL conducted the bioinformatics analysis, interpreted the data, and optimized the graphs. SW and HC prepared and wrote the manuscript. SW, Y-WT, and WD revised and approved the final manuscript. All authors contributed to the article and approved the submitted version.

## Funding

This work was supported by Shenzhen High-level Hospital Construction Fund (YBH2019-260), Shenzhen Key Medical Discipline Construction Fund (No. SZXK027), Sanming Project of Medicine in Shenzhen (No. SZSM202011016), and Scientific Research Foundation of PEKING UNIVERSITY SHENZHEN HOSPITAL (No. KYQD2021075).

## Conflict of Interest

Author SW was employed by company Tianjin KingMed Center for Clinical Laboratory Co. Ltd.

The remaining authors declare that the research was conducted in the absence of any commercial or financial relationships that could be construed as a potential conflict of interest.

## Publisher’s Note

All claims expressed in this article are solely those of the authors and do not necessarily represent those of their affiliated organizations, or those of the publisher, the editors and the reviewers. Any product that may be evaluated in this article, or claim that may be made by its manufacturer, is not guaranteed or endorsed by the publisher.

## Supplementary Material

Supplementary Table S1 The relative abundance of phylum and genera identified in all samples.

## References

[B1] Barichello T. Sayana P. Giridharan V. V. Arumanayagam A. S. Narendran B. Della Giustina A.. (2019). Long-Term Cognitive Outcomes After Sepsis: A Translational Systematic Review. Mol. Neurobiol. 56 (1), 186–251. doi: 10.1007/s12035-018-1048-2 29687346

[B2] Cecconi M. Evans L. Levy M. Rhodes A. (2018). Sepsis and Septic Shock. Lancet 392 (10141), 75–87. doi: 10.1016/s0140-6736(18)30696-2 29937192

[B3] Coopersmith C. M. De Backer D. Deutschman C. S. Ferrer R. Lat I. Machado F. R.. (2018). Surviving Sepsis Campaign: Research Priorities for Sepsis and Septic Shock. Intensive Care Med. 44 (9), 1400–1426. doi: 10.1007/s00134-018-5175-z 29971592 PMC7095388

[B4] Cui L. Guan X. Ding W. Luo Y. Wang W. Bu W.. (2021). Scutellaria Baicalensis Georgi Polysaccharide Ameliorates DSS-Induced Ulcerative Colitis by Improving Intestinal Barrier Function and Modulating Gut Microbiota. Int. J. Biol. Macromolecules 166, 1035–1045. doi: 10.1016/j.ijbiomac.2020.10.259 33157130

[B5] Cui H.-X. Zhang L.-S. Luo Y. Yuan K. Huang Z.-Y. Guo Y. (2019). A Purified Anthraquinone-Glycoside Preparation From Rhubarb Ameliorates Type 2 Diabetes Mellitus by Modulating the Gut Microbiota and Reducing Inflammation. Front. Microbiol. 10, 1423. doi: 10.3389/fmicb.2019.01423 31293553 PMC6603233

[B6] Haak B. W. Prescott H. C. Wiersinga W. J. (2018). Therapeutic Potential of the Gut Microbiota in the Prevention and Treatment of Sepsis. Front. Immunol. 9, 2042. doi: 10.3389/fimmu.2018.02042 30250472 PMC6139316

[B7] Haak B. W. Wiersinga W. J. (2017). The Role of the Gut Microbiota in Sepsis. Lancet Gastroenterol. Hepatol. 2 (2), 135–143. doi: 10.1016/s2468-1253(16)30119-4 28403983

[B8] Hua Y. L. Jia Y. Q. Zhang X. S. Yuan Z. W. Ji P. Hu J. J.. (2021). Baitouweng Tang Ameliorates DSS-Induced Ulcerative Colitis Through the Regulation of the Gut Microbiota and Bile Acids *via* Pathways Involving FXR and TGR5. BioMed. Pharmacother. 137, 111320. doi: 10.1016/j.biopha.2021.111320 33578232

[B9] Jia Q. Wang L. Zhang X. Ding Y. Li H. Yang Y.. (2020). Prevention and Treatment of Chronic Heart Failure Through Traditional Chinese Medicine: Role of the Gut Microbiota. Pharmacol. Res. 151, 104552. doi: 10.1016/j.phrs.2019.104552 31747557

[B10] Ji C. Deng Y. Yang A. Lu Z. Chen Y. Liu X.. (2020). Rhubarb Enema Improved Colon Mucosal Barrier Injury in 5/6 Nephrectomy Rats May Associate With Gut Microbiota Modification. Front. Pharmacol. 11, 1092. doi: 10.3389/fphar.2020.01092 32848732 PMC7403201

[B11] Kim M. H. Choi J. H. (2020). An Update on Sepsis Biomarkers. Infect. Chemother. 52 (1), 1–18. doi: 10.3947/ic.2020.52.1.1 32239808 PMC7113456

[B12] Komatsu S. Sakamoto E. Norimizu S. Shingu Y. Asahara T. Nomoto K.. (2016). Efficacy of Perioperative Synbiotics Treatment for the Prevention of Surgical Site Infection After Laparoscopic Colorectal Surgery: A Randomized Controlled Trial. Surg. Today 46 (4), 479–490. doi: 10.1007/s00595-015-1178-3 25933911

[B13] Lankelma J. M. van Vught L. A. Belzer C. Schultz M. J. van der Poll T. de Vos W. M.. (2017). Critically Ill Patients Demonstrate Large Interpersonal Variation in Intestinal Microbiota Dysregulation: A Pilot Study. Intensive Care Med. 43 (1), 59–68. doi: 10.1007/s00134-016-4613-z 27837233 PMC5203863

[B14] Lewis-Mikhael A.-M. Davoodvandi A. Jafarnejad S. (2020). Effect of Lactobacillusplantarum Containing Probiotics on Blood Pressure: A Systematic Review and Meta-Analysis. Pharmacol. Res. 153, 104663. doi: 10.1016/j.phrs.2020.104663 31987993

[B15] Liu D. Q. Gao Q. Y. Liu H. B. Li D. H. Wu S. W. (2013a). Probiotics Improve Survival of Septic Rats by Suppressing Conditioned Pathogens in Ascites. World J. Gastroenterol. 19 (25), 4053–4059. doi: 10.3748/wjg.v19.i25.4053 23840152 PMC3703194

[B16] Liu D. Q. Gao Q. Y. Wu S. W. Li D. H. Liu H. B. (2013b). Pharmocodynamic Observation Qingre Jiedu Decoction Improve Survival on Rats Experimental Sepsis. Chin. J. Exp. Traditional Med. Formulae 19 (16), 246–249. doi: 10.11653/syfj2013160246

[B17] Li W. Wu X. Hu X. Wang T. Liang S. Duan Y.. (2017). Structural Changes of Gut Microbiota in Parkinson's Disease and its Correlation With Clinical Features. Sci. China Life Sci. 60 (11), 1223–1233. doi: 10.1007/s11427-016-9001-4 28536926

[B18] Ojima M. Motooka D. Shimizu K. Gotoh K. Shintani A. Yoshiya K.. (2016). Metagenomic Analysis Reveals Dynamic Changes of Whole Gut Microbiota in the Acute Phase of Intensive Care Unit Patients. Dig. Dis. Sci. 61 (6), 1628–1634. doi: 10.1007/s10620-015-4011-3 26715502 PMC4875048

[B19] Oniszczuk A. Oniszczuk T. Gancarz M. Szymanska J. (2021). Role of Gut Microbiota, Probiotics and Prebiotics in the Cardiovascular Diseases. Molecules 26 (4), 1172. doi: 10.3390/molecules26041172 33671813 PMC7926819

[B20] Paoli C. J. Reynolds M. A. Sinha M. Gitlin M. Crouser E. (2018). Epidemiology and Costs of Sepsis in the United States-An Analysis Based on Timing of Diagnosis and Severity Level. Crit. Care Med. 46 (12), 1889–1897. doi: 10.1097/CCM.0000000000003342 30048332 PMC6250243

[B21] Prescott H. C. Dickson R. P. Rogers M. A. Langa K. M. Iwashyna T. J. (2015). Hospitalization Type and Subsequent Severe Sepsis. Am. J. Respir. Crit. Care Med. 192 (5), 581–588. doi: 10.1164/rccm.201503-0483OC 26016947 PMC4595694

[B22] Shin N. R. Whon T. W. Bae J. W. (2015). Proteobacteria: Microbial Signature of Dysbiosis in Gut Microbiota. Trends Biotechnol. 33 (9), 496–503. doi: 10.1016/j.tibtech.2015.06.011 26210164

[B23] Taft D. H. Ambalavanan N. Schibler K. R. Yu Z. Newburg D. S. Deshmukh H.. (2015). Center Variation in Intestinal Microbiota Prior to Late-Onset Sepsis in Preterm Infants. PLoS One 10 (6), e0130604. doi: 10.1371/journal.pone.0130604 26110908 PMC4482142

[B24] Takiishi T. Fenero C. I. M. Camara N. O. S. (2017). Intestinal Barrier and Gut Microbiota: Shaping Our Immune Responses Throughout Life. Tissue Barriers 5 (4), e1373208. doi: 10.1080/21688370.2017.1373208 28956703 PMC5788425

[B25] Tong X. Xu J. Lian F. Yu X. Zhao Y. Xu L.. (2018). Structural Alteration of Gut Microbiota During the Amelioration of Human Type 2 Diabetes With Hyperlipidemia by Metformin and a Traditional Chinese Herbal Formula: A Multicenter, Randomized, Open Label Clinical Trial. mBio 9 (3), e02392–17. doi: 10.1128/mBio.02392-17 29789365 PMC5964358

[B26] Toscano M. De Grandi R. Miniello V. L. Mattina R. Drago L. (2017). Ability of Lactobacillus Kefiri LKF01 (DSM32079) to Colonize the Intestinal Environment and Modify the Gut Microbiota Composition of Healthy Individuals. Dig. Liver Dis. 49 (3), 261–267. doi: 10.1016/j.dld.2016.11.011 27939319

[B27] Wan Y. D. Zhu R. X. Wu Z. Q. Lyu S. Y. Zhao L. X. Du Z. J.. (2018). Gut Microbiota Disruption in Septic Shock Patients: A Pilot Study. Med. Sci. Monit. 24, 8639–8646. doi: 10.12659/MSM.911768 30488879 PMC6282651

[B28] Wu D. Ding L. Tang X. Wang W. Chen Y. Zhang T. (2019). Baicalin Protects Against Hypertension-Associated Intestinal Barrier Impairment in Part Through Enhanced Microbial Production of Short-Chain Fatty Acids. Front. Pharmacol. 10, 1271. doi: 10.3389/fphar.2019.01271 31719823 PMC6826474

[B29] Yu L. Xing Z. K. Mi S. L. Wu X. (2019). Regulatory Effect of Traditional Chinese Medicine on Intestinal Microbiota. Zhongguo Zhong Yao Za Zhi 44 (1), 34–39. doi: 10.19540/j.cnki.cjcmm.20181101.013 30868809

[B30] Zhang S. Chen D. C. (2019). Facing a New Challenge: The Adverse Effects of Antibiotics on Gut Microbiota and Host Immunity. Chin. Med. J. (Engl.) 132 (10), 1135–1138. doi: 10.1097/CM9.0000000000000245 30973451 PMC6511407

